# Clinical Characteristics and Mortality of Neurocritical Care Patients With Post-traumatic Cerebral Venous Outflow Compromise

**DOI:** 10.1227/neuprac.0000000000000145

**Published:** 2025-06-24

**Authors:** Tommi K. Korhonen, Moritz Steinruecke, David Clark, Ivan Timofeev, Adel Helmy, Andrea Lavinio, Nicholas J. Higgins, John Pickard, Peter Hutchinson, Angelos Kolias

**Affiliations:** * Division of Neurosurgery, Department of Clinical Neurosciences, Cambridge University Hospitals NHS Foundation Trust & University of Cambridge, Cambridge, Cambridgeshire, UK;; ‡ Department of Anaesthesia, Cambridge University Hospitals NHS Foundation Trust, Cambridge, Cambridgeshire, UK;; § Department of Radiology, Cambridge University Hospitals NHS Foundation Trust, Cambridge, Cambridgeshire, UK

## Abstract

**BACKGROUND AND OBJECTIVES:**

The clinical characteristics and natural history of post-traumatic cerebral venous outflow compromise (VOC) are poorly characterized. We aimed to determine the prevalence of VOC in at-risk traumatic brain injury patients, assess its effect on long-term mortality, and describe our management practices.

**METHODS:**

We conducted a retrospective single-center case-control study. We included patients admitted to the neurocritical care unit after traumatic brain injury who had been investigated with computed tomography venography because of clinical suspicion of VOC between 2008 and 2015.

**RESULTS:**

A total of 89 patients underwent computed tomography venography during their neurocritical care unit admission. A total of 43 patients (48%, 32 male [74%], mean age 40 years [SD 16.2]) had evidence of VOC. Of these, 28 (65%) were due to an intraluminal dural venous sinus thrombosis (DVST) and 15 (35%) due to an extraluminal cause. Twelve (43%) of the DVSTs were occlusive, and 16 (57%) were nonocclusive. A total of 24 patients (27%) underwent decompressive craniectomy, which was more commonly performed for patients with an occlusive thrombosis than those with partial or no VOC (67% vs 23% vs 20%, *P* < .01). A total of 4 patients (14%) with an intraluminal thrombosis received antithrombotic therapy. Mortality rate of those with VOC may have been higher compared with those without VOC at 14 days (14% vs 7%, *P* = .31) but was similar at 5 years (21% vs 22%, *P* = .93). Patients with a midline or bilateral thrombosis had higher overall mortality (83% vs 18%, *P* = .01) than those with a thrombosis located elsewhere.

**CONCLUSION:**

Among those at risk, patients with evidence of post-traumatic VOC may have had higher short-term mortality, but VOCs did not increase long-term mortality rates compared with those without VOC. Patients with an occlusive thrombosis were more likely to undergo decompressive craniectomy. Most patients with a DVST received prophylactic rather than treatment-dose antithrombosis. Further studies are required to determine the optimal management of post-traumatic VOC.

**ABBREVIATIONS:**
CTV: computed tomography venography
DC: decompressive craniectomy
DVST: dural venous sinus thrombosis
EVD: external ventricular drainage
LMWH: low molecular weight heparin
NCCU: neurocritical care unit
TBI: traumatic brain injury
VOC: venous outflow compromise.

Traumatic brain injury (TBI) may cause venous outflow compromise (VOC).^1-6^ This is most commonly due to dural venous sinus thrombosis (DVST) or extraluminal compression from a depressed skull fracture or a hematoma. Cerebral computed tomography venography (CTV) is the diagnostic imaging modality of choice. The main indication for CTV in the context of TBI is the presence of a skull fracture crossing a dural venous sinus or involving the petrous portion of the temporal bone. In addition, an unexplained clinical deterioration or rise in intracranial pressure (ICP) after head injury may warrant investigation with CTV once other causes have been excluded.

Although the natural history of nontraumatic VOC is well characterized,^7,8^ there is less evidence on the clinical characteristics and outcomes of patients with post-traumatic VOC. Moreover, treatments typically used for nontraumatic DVST, such as anticoagulation, may not be appropriate in trauma patients because of the risk of exacerbating preexisting hemorrhage.^9^ In this study, we aimed to:

1. Determine the prevalence of post-traumatic VOC in at-risk TBI patients in our neurocritical care unit (NCCU),
2. Assess the effect of post-traumatic VOC on short-term and long-term mortality, and
3. Examine our practice of managing these patients.

## METHODS

### Patient Selection and Study Setting

This was an observational study which included all patients admitted to Cambridge University Hospital NCCU after a TBI and underwent CTV because of clinical suspicion of VOC during their acute admission between January 2008 and December 2015 (catchment population approximately 6 000 000 people). The decision to obtain a CTV was made by the clinical team based on suspicion of post-traumatic VOC, either due to the patient sustaining a skull fracture crossing a dural venous sinus or an unexplained clinical deterioration, such as an unexplained increase in ICP >20 to 25 mm Hg despite management according to international TBI guidelines^10^ in a stepwise manner.^11^ Prophylaxis for deep vein thrombosis involved the application of pneumatic compression boots from the day of admission and commencement of prophylactic low molecular weight heparin (LMWH) typically within 48 to 72 hours, provided there was no evidence of contraindications.

Patients who received CTV for indications other than diagnosing post-traumatic VOC were excluded. Demographical, clinical, and radiological parameters and treatments were extracted from the electronic medical records. Mortality data were queried from the UK National Health Service Spine portal, which captures all mortality within the United Kingdom. The Corticosteroid Randomization After Significant Head Injury (CRASH) trial calculator was used to calculate predicted 14-day mortality.^12^

### Imaging and Interpretation

If required, a plain computed tomography head scan was obtained, followed by intravenous administration of an iodinated contrast agent. Once the contrast agent reached a sufficient concentration in the dural venous sinuses, a thin-sliced computed tomography was obtained from skull base to vertex. The dominance of either transverse sinus was noted. A sinus filling defect, possibly accompanied by gyral enhancement and cerebral venous prominence, suggested the presence of DVST. In the presence of a skull fracture or hematoma, venous sinus compression may have resulted in focally narrowed opacification of the affected sinus. All scans were reviewed by a radiology registrar and reported by a neuroradiology consultant. For this study, scans and reports were retrospectively reviewed by 2 neurosurgeons (D.C., A.K.). Disputes in interpretation were resolved by discussion with a neuroradiologist (N.J.H.).

### Statistical Analysis

All statistical analyses were performed using SPSS Version 23.0 (IBM Corp). The Pearson χ^2^ and Fisher exact tests were used to compare categorical variables across groups. Analysis of variance was used to compare group distributions. Mean values are reported with SDs and median values with IQR. A *P*-value of <0.05 was considered statistically significant. The degree of VOC was categorized as no VOC, partial VOC, and complete VOC. The partial VOC group comprised patients with nonocclusive DVST, extrinsic compression, eg due to a depressed skull fracture, or displacement due to a hematoma, of a dural venous sinus. The complete VOC group comprised patients with occlusive DVST. For mortality analyses, the follow-up time was calculated as the time from injury to death or December 2022. The Strengthening the Reporting of Observational Studies in Epidemiology (STROBE) guidelines were adhered to.

### Ethical Considerations

This study was part of a service evaluation on the management of post-traumatic VOC. The protocol (PRN11024) was approved by the Cambridge University Hospitals NHS Foundation Trust Audit Committee and the Neurosurgery Audit Lead. Ethical review board evaluation was waived because of the retrospective study setting, and patient consent was not required for the same reason.

## RESULTS

### Baseline Data

**FIGURE 1.**
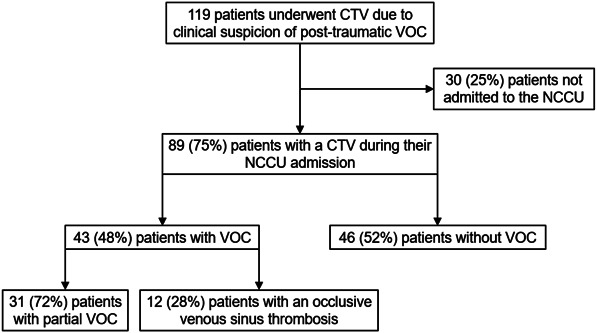
Flowchart outlining the inclusion process of patients who underwent cerebral CTV because of suspicion of post-traumatic VOC. CTV, CT venography; NCCU, neurocritical care unit; VOC, venous outflow compromise.

Eighty-nine TBI patients with a mean of 41.7 years (SD 16.4) were admitted to the NCCU and underwent CTV (Figure 1). Seventy patients (79%) were male, and 6 patients (7%) were younger than 18 years. The mean follow-up time was 94.5 months (SD 49.0).

### Radiological Findings

**FIGURE 2.**
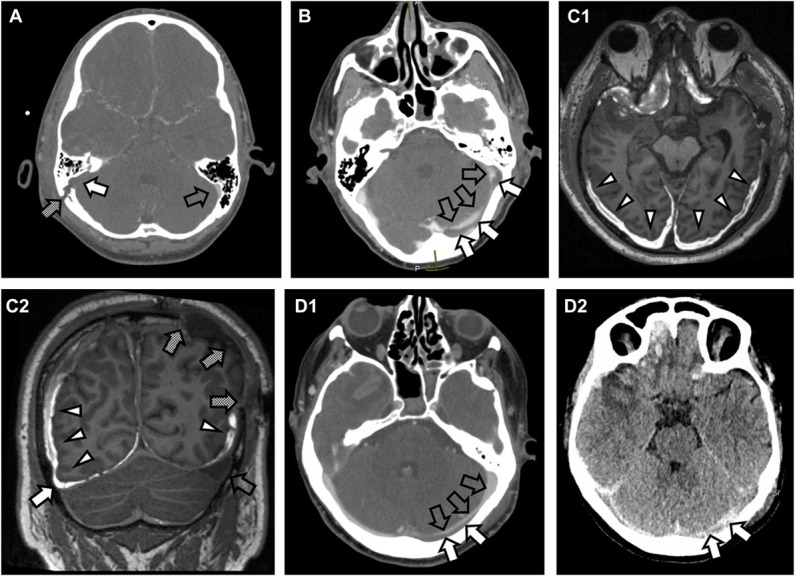
Representative scans of different intracranial pathologies causing post-traumatic VOC. **A**, A CTV showing absence of contrast in the right sigmoid sinus (white arrow) adjacent to a skull fracture (shaded arrow), suggestive of an occlusive sinus thrombosis. Contrast is present in the left sigmoid sinus (transparent arrow). **B**, A CTV of incomplete contrast enhancement of the left transverse and sigmoid sinuses (transparent arrows) associated with a low-density filling defect in the transverse sinus (white arrows) associated with a skull fracture crossing the sinus, suggestive of a nonocclusive sinus thrombosis. **C1**, A semiaxial gadolinium contrast-enhanced T1-weighted magnetic resonance scan showing subdural hematomas (white arrowheads). **C2**, The subdural hematomas cause narrowing of the nondominant right transverse sinus (white arrow), suggestive of extrinsic compression of the sinus. The dominant left transverse sinus (transparent arrow) has expanded because of an ipsilateral decompressive craniectomy (shaded arrows) and the right-sided VOC. **D1**, A CTV of narrowed contrast-enhancement of the left transverse sinus (transparent arrows) because of a thin skull fracture–associated epidural collection (white arrows) displacing the sinus away from the inner table of the skull without fully occluding the sinus. **D2**, The epidural collection (white arrows) is better visualized in the non–contrast-enhanced CT scan. CT, computed tomography; CTV, CT venography; VOC, venous outflow compromise.

The median time from injury to CTV was 2.0 days (IQR 1.0-4.0). Patients with VOC underwent their initial CTV earlier than those without VOC (median 1.0 day [IQR 1.0-3.0] vs median 3.0 days [IQR 1.0-6.3], *P* = .003). Of the 43 patients with VOC, the compromise was caused by occlusive DVST in 12 (28%), nonocclusive DVST in 16 (37%), sinus displacement due to fracture or hematoma in 9 (21%), and external compression due to fracture or hematoma in 6 cases (14%) (Figure 2). Clinical characteristics were similar between patients with and without VOC (Table 1). Seventy-nine patients (89%) had a skull fracture crossing a venous sinus. Of these patients, 41 (52%) had radiological evidence of VOC on CTV. There were no significant differences in the worst acute-stage Marshall grades between the VOC and no VOC groups (Table 2).

**TABLE 1.** Clinical Characteristics of the 89 TBI Patients Admitted to the NCCU Who Underwent a CT Venogram Due to Suspicion of Venous Outflow Compromise

Characteristic	VOC (n = 43)	No VOC (n = 46)	*P*-value
Mean age at injury, y (SD)	40.3 (16.2)	43.1 (16.6)	.41
Sex, n (%)			.44
Male	32 (74)	38 (83)	
Female	11 (26)	8 (17)	
Admission GCS, n (%)^a^			.06
13-15	6 (15)	3 (7)	
9-12	3 (8)	11 (27)	
3-8	30 (77)	27 (59)	
Admission reactivity of pupils, n (%)			.89
Both	34 (87)	38 (84)	
One	3 (8)	3 (7)	
None	2 (5)	4 (9)	
Extracranial injury, n (%)			.99
Yes	21 (49)	22 (48)	
No	22 (51)	24 (52)	
Craniotomy for mass lesion, n (%)			.24
Yes	13 (30)	9 (20)	
No	30 (70)	37 (80)	
External ventricular drain, n (%)			.50
Yes	12 (28)	10 (22)	
No	31 (72)	36 (78)	
Decompressive craniectomy, n (%)			.10
Yes	15 (35)	9 (20)	
No	28 (65)	37 (80)	
Mean NCCU stay length, d (SD)	17.1 (11.2)	18.0 (12.8)	.72
CRASH 14-d expected mortality, n (%)^b^	6 (17)	7 (18)	.83
Observed 14-d mortality, n (%)	6 (14)	3 (7)	.31
Observed 30-d mortality, n (%)	7 (16)	5 (11)	.54
Observed 5-y mortality, n (%)	9 (21)	10 (22)	.93
Observed overall mortality, n (%)	15 (35)	14 (30)	.82

CRASH, Corticosteroid Randomization After Significant Head Injury; GCS, Glasgow Coma Scale; NCCU, neurocritical care unit; VOC, venous outflow compromise.

a Data available for 80 patients (90%) (39 [91%] and 41 [89%] in the VOC and no VOC groups, respectively).

b Data available for 76 patients (85%) (36 [84%] and 40 [87%] in the VOC and no VOC groups, respectively).

Patients are stratified based on the presence or absence of VOC.

**TABLE 2.** Marshall Grades of the 89 Traumatic Brain Injury Patients Who Underwent Computed Tomography Venography Due to Clinical Suspicion of a Cerebral VOC

Marshall grade	VOC (n = 43) (%)	No VOC (n = 46) (%)	*P*-value
Grade 1: No visible pathology	0 (0)	1 (2)	.60
Grade 2: Mixed density lesions, shift <6 mm, cisterns present	22 (51)	28 (61)	
Grade 3: Cisterns compressed, shift <6 mm	6 (14)	3 (7)	
Grade 4: Cisterns compressed, shift >5 mm	3 (7)	3 (7)	
Grade 5: Evacuated mass lesion	12 (28)	10 (22)	
Grade 6: Nonevacuated mass lesion >25 cc	0 (0)	1 (2)	

VOC, venous outflow compromise.

VOC was more commonly identified in the lateral sinuses (29/43 [67%]) than the superior sagittal sinus (SSS, 8/43 [19%]) (**Supplemental Digital Content 1**, http://links.lww.com/NS9/A51). DVST of the lateral sinuses was more commonly occlusive when the thrombus was on the right (10/14 [71%]) compared with the left (2/7 [29%]) (**Supplemental Digital Content 1**, http://links.lww.com/NS9/A51). At median, 11 (IQR 4.5-19) CTV scans were performed per year after head injuries, and a median of 197 (IQR 181-234) patients were admitted per year to the NCCU because of a head injury.

### Management

External ventricular drainage (EVD) and decompressive craniectomy (DC) were conducted as commonly among those with VOC as those without VOC (Table 1). Patients with complete VOC were more likely to undergo DC than those with partial or no VOC (8/12 [67%] vs 7/31 [23%] vs 9/46 [20%], respectively, *P* = .007). Patients who underwent DC had more severe TBIs than those who did not (mean Marshall grade 3.8 [SD 1.3] vs 2.7 [SD 1.2], respectively, *P* < .001). There was no difference in the proportion of patients with an EVD between the complete, partial, and no VOC groups (5/12 [42%] vs 7/31 [23%] vs 10/46 [22%], respectively, *P* = .34).

Reliable medication data were available for patients admitted after July 2014 (n = 18). Of them, 15 (83%) had received prophylactic LMWH in accordance with our institutional protocol. This included 3 patients with nonocclusive DVSTs and 1 with an occlusive DVST. Three of these patients (1 occlusive DVST and 2 of the nonocclusive DVSTs) had received prophylactic LMWH, and 2 of the nonocclusive thromboses were managed with treatment-level dosing (1 with warfarin and 1 with heparin infusion).

## OUTCOMES FOLLOWING VENOUS OUTFLOW COMPROMISE

In the present cohort, the 14-day, 30-day, 5-year, and overall mortality rates were 10% (n = 9), 13% (n = 12), 23% (n = 19), and 33% (n = 29), respectively. Patients with VOC may have higher 14-day mortality than those without VOC, but this did not reach statistical significance, and long-term mortality and length of NCCU stay were comparable (Table 1).

Mortality rates and length of NCCU stay were similar between patients with complete and partial VOC (Table 3). Patients with a complete VOC had higher 14-day mortality than those with partial or no VOC, but the difference was not statistically significant (3/12 [25%] vs 3/31 [10%] vs 3/46 [7%], respectively, *P* = .17). Five-year mortality rates were also similar between these groups (complete VOC 3/12 [25%], partial VOC 6/31 [19%], no VOC 10/46 [22%]; *P* = .92). 14-day mortality was similar between patients with a DVST and those with extraluminal sinus compression (5/28 [18%] vs 1/15 [7%], respectively, *P* = .40), as was 5-year mortality (7/28 [25%] vs 2/15 [13%], respectively, *P* = .46).

**TABLE 3.** Effect of Occlusion of Sinus on Mortality and Length of Stay in the NCCU

Variable	Complete VOC (n = 12)	Partial VOC (n = 31)	*P*-value
14-d mortality, n (%)	3 (25)	3 (10)	.33
5-y mortality, n (%)	3 (25)	6 (19)	.69
Mean NCCU stay length, d (SD)	18.9 (9.0)	16.5 (12.0)	.52

NCCU, neurocritical care unit; VOC, venous outflow compromise.

Patients with a midline or bilateral DVST had higher overall mortality than those with thromboses in other locations (Table 4), although these were all nonocclusive (**Supplemental Digital Content 1**, http://links.lww.com/NS9/A51).

**TABLE 4.** Effect of DVST Location on Mortality

Variable	Midline or bilateral DVST (n = 6)	Other DVST location (n = 22)	*P*-value
14-d mortality, n (%)	2 (33)	3 (14)	.29
30-d mortality, n (%)	3 (50)	3 (14)	.09
5-y mortality, n (%)	3 (50)	4 (18)	.14
Overall mortality, n (%)	5 (83)	4 (18)	.01

DVST, dural venous sinus thrombosis.

### Complications of VOC

One patient (1% of total, 4% of those with DVST) developed a venous infarct. The patient had radiological evidence of right transverse and sigmoid sinus thrombosis and corticosubcortical hypodensity consistent with infarction in the right parietal lobe. At most recent follow-up, his extended Glasgow Outcome Scale-Extended was 5.

### Follow-Up of DVST

Of the 12 patients with occlusive DVSTs, 2 (17%) had undergone a repeat CTV postoperatively, at 30 and 164 days, and neither had recanalized. Eight of 16 patients (50%) with nonocclusive DVSTs had follow-up CTV scans, of whom 1 (13%) had partial and 1 (13%) had total recanalization of the thrombotic sinus. Six (75%) showed no changes in their DVST. These scans were performed at a mean time of 45 (SD 98) days after the initial CTV.

## DISCUSSION

### Outcomes

This study was the first to evaluate outcomes beyond 5 years in patients with post-traumatic VOC. VOC was not associated with increased long-term mortality but may have slightly increased the 14-day mortality rate in our study. Some previously published studies suggest increased inpatient mortality among patients with post-traumatic VOC, but most of the evidence to date has concentrated on short-term outcomes.^13^

Patients with occlusive VOC may have had higher short-term mortality than those with partially occlusive or no VOC (25% vs 8%, respectively), but this difference was not statistically significant, and 5-year mortality rates were comparable (25% vs 21%, respectively) between the groups. Benifla et al observed a trend toward worse functional outcomes in patients with occlusive compared with partial VOC,^3^ but we did not find such an effect on mortality. In our study, patients with midline or bilateral DVST had higher overall mortality than those with DVST located elsewhere, corroborating the findings of Netteland et al.^14^ This difference may be due to the DVST altering the venous clearance of both cerebral hemispheres: A unilateral DVST is unlikely to disturb the global venous drainage if the contralateral sinuses remain patent. Interestingly, none of the bilateral DVSTs or those situated in the SSS were fully occlusive despite resulting in increased mortality in our study. In our overall TBI cohort, we found that mortality increased from 10% at 14 days to 33% at the mean follow-up time of 94.5 months. This is in line with previous literature demonstrating significant long-standing excess mortality after TBI.^15,16^

### Radiological Findings

The main indications for venography in the context of head trauma are a skull fracture, with a higher suspicion for VOC if the fracture crosses a venous sinus or the jugular bulb, and unexplained refractory intracranial hypertension.^13,17^ Forty-eight percent of patients undergoing CTV because of clinical suspicion of VOC after TBI had evidence of VOC, 65% of whom had a DVST. Correspondingly, the incidence of VOC in patients imaged because of clinical suspicion ranges between 22% and 51% in the literature.^13^

Ten patients (11%) who underwent a CTV did not have a skull fracture, and two (20%) of these patients had VOCs, both nonocclusive DVSTs. The incidence of VOC among all TBI patients is unclear, but Bokhari et al^17^ suggested that DVSTs occur in 4% of all TBI patients.

Consistent with previous reports,^3^ we observed that the lateral sinuses were more commonly affected by VOC than the SSS. Right-sided DVSTs were more likely to be occlusive than left-sided thromboses. The right transverse sinus is more commonly dominant than the left,^18^ but it is unclear whether there is an association between sinus dominance and thrombotic occlusion.

### Surgical Management

#### External Ventricular Drain

VOCs increase ICP through reduced venous outflow and cerebrospinal fluid (CSF) absorption.^19^ EVD insertion reduces ICP by reducing CSF volume, which could be particularly useful in patients with VOC-related increases in ICP. Indeed, placement of a CSF shunt has been reported for raised ICP in the context of post-traumatic VOC,^20^ although the benefit of CSF diversion is not well established in nontraumatic cerebral venous thrombosis,^21^ and the insertion of an EVD may be challenging because of compensatorily reduced CSF volume in the context of raised ICP. Nevertheless, EVD insertion is an important step in the tiered management of TBI.^11^

#### Craniotomy

VOC may be caused by direct pressure on the sinus because of an intracranial hematoma or a depressed skull fracture (Figure 2). Surgical management of these lesions may be performed to reduce mass effect and/or treat the VOC. Suturing the lacerated sinus may be required, but procedures should be balanced against the risks of catastrophic blood loss and air embolism.^22^ Surgical thrombectomy and local thrombolysis have been suggested in previous case reports in the treatment of severe nontraumatic cerebral venous thrombosis.^23,24^ No such procedures had been conducted in our cohort, but in cases of sinus displacement because of mass lesions, craniotomies and DCs may have alleviated the VOC by removing the mass lesion causing venous compression.

#### Decompressive Craniectomy

Patients with occlusive DVST were more likely to undergo DC than those with partial or no VOC. This may have affected the observed outcomes in our study and possibly diminished the mortality differences described previously between those with and without post-traumatic DVST.^4,25^ Clinically, the increased need for DC may reflect an effect of DVST on ICP and possibly supports DC as a management option, although the patients who underwent DC had radiologically more severe brain injuries than those who did not. DC has been used in nontraumatic DVST,^7^ and the American Stroke Association guidelines support consideration of DC in cases of intractable ICP because of spontaneous cerebral venous thrombosis,^26^ but evidence on DC after trauma-related VOC is scant. Importantly, the mechanism of action of DC after DVST is unclear—DC increases cerebral perfusion,^27^ and consequently venous outflow, which in the case of an obstructed sinus may be problematic. Furthermore, the increased surgical complexity and risk of postcraniectomy hematomas because of anticoagulation must be considered when undertaking DC. Last, although our TBI management protocol is ICP and cerebral perfusion pressure–guided,^11^ the diagnosis of DVST may bias clinical decision making toward DC, which could partially explain the differing operation rates between the groups. We were unable to assess this effect in more detail because of a lack of comprehensive ICP data.

### Medical Therapy

A total of 4 of 28 patients (14%) with DVST received treatment-dose antithrombosis in the acute phase. Mechanical and medical prophylaxis for deep venous thrombosis was commenced routinely in the absence of contraindications. Antithrombotic protocols for post-traumatic VOC vary in the literature; Wang et al treated all of their VOC patients with urokinase, Netteland et al administered LMWH to 82% of their cerebral venous thrombosis patients, Harris et al treated 13% of their DVST patients with anticoagulation or antiplatelets, and Fujii et al treated 5% of their VOC patients with heparin.^4,14,28,29^ In the context of spontaneous DVST, anticoagulation is considered safe and effective.^30-32^ Commonly accepted guidelines recommend anticoagulation even in the presence of a secondary intracerebral hematoma.^26,33^ Extrapolating this to trauma-related indications is challenging, particularly with the tendency of traumatic hematomas to increase in size after the initial injury.^9,34,35^ Benifla et al proposed a protocol for the diagnosis and management of post-traumatic DVST based on their experience in managing 21 post-traumatic VOC patients. They recommend anticoagulation in patients with a complete DVST, except in the presence of an intracranial hematoma.^3^ In our center, most TBI patients are started on a prophylactic dose of LMWH once the traumatic intracranial lesions have stabilized in follow-up imaging.

The challenges with anticoagulation in post-traumatic DVST raise the prospect of endovascular treatment. Options include mechanical thrombectomy, intravascular thrombolysis, or a combination of these approaches.^36,37^ They may be considered in occlusive DVST which fails to respond to systemic anticoagulation. The recent Thrombolysis or Anticoagulation for Cerebral Venous Thrombosis (TO-ACT) trial demonstrated similar mortality and functional outcome between endovascular management and anticoagulation after spontaneous DVST.^8^ Still, post-traumatic VOC is commonly complicated by extraluminal factors, and the baseline characteristics of TBI patients are very different from those in the TO-ACT trial. Currently, there are no robust guidelines or high-level evidence for the management of trauma-related VOC, and its management has remained largely unchanged since the study period.

### Complications of Cerebral VOC

In our cohort, 1 patient (4%) with DVST developed a venous infarct, which is slightly lower than the complication rates in the existing literature. Delgado et al observed venous infarcts in 7% of post-traumatic DVST patients and Netteland et al in 18%.^2,14^ Such differences may be caused by differences in the diagnostic pathways and follow-up protocols.

### Limitations

The conclusions of this study are limited by its retrospective methodology. We were unable to collect comprehensive data on recanalization rates and functional outcomes apart from mortality. Nevertheless, we were able to produce previously missing information on long-term survival of patients with VOC after TBI.

## CONCLUSIONS

Among those at risk, patients with evidence of post-traumatic VOC may have had higher short-term mortality but VOCs did not increase long-term mortality rates compared with those without VOC. Patients with an occlusive thrombosis were more likely to undergo DC. Most patients with an intraluminal thrombosis received prophylactic rather than treatment-dose antithrombosis. Further studies are required to determine the optimal management of post-traumatic VOC.

## SUPPLEMENTAL DIGITAL CONTENT

**Supplemental Digital Content 1. Table 1.** Site of venous outflow compromise stratified by the type of venous compression on CT venography.
